# Information-Theoretic Analysis of the Dynamics of an Executable Biological Model

**DOI:** 10.1371/journal.pone.0059303

**Published:** 2013-03-19

**Authors:** Avital Sadot, Septimia Sarbu, Juha Kesseli, Hila Amir-Kroll, Wei Zhang, Matti Nykter, Ilya Shmulevich

**Affiliations:** 1 Institute for Systems Biology, Seattle, Washington, United States of America; 2 Department of Signal Processing, Tampere University of Technology, Tampere, Finland; 3 Department of Medicine I, University Medical Centre Hamburg-Eppendorf, Hamburg, Germany; 4 Department of Pathology, The University of Texas M. D. Anderson Cancer Center, Houston, Texas, United States of America; 5 Institute of Biomedical Technology, University of Tampere, Tampere, Finland; Indiana University, United States of America

## Abstract

To facilitate analysis and understanding of biological systems, large-scale data are often integrated into models using a variety of mathematical and computational approaches. Such models describe the dynamics of the biological system and can be used to study the changes in the state of the system over time. For many model classes, such as discrete or continuous dynamical systems, there exist appropriate frameworks and tools for analyzing system dynamics. However, the heterogeneous information that encodes and bridges molecular and cellular dynamics, inherent to fine-grained molecular simulation models, presents significant challenges to the study of system dynamics. In this paper, we present an algorithmic information theory based approach for the analysis and interpretation of the dynamics of such executable models of biological systems. We apply a normalized compression distance (NCD) analysis to the state representations of a model that simulates the immune decision making and immune cell behavior. We show that this analysis successfully captures the essential information in the dynamics of the system, which results from a variety of events including proliferation, differentiation, or perturbations such as gene knock-outs. We demonstrate that this approach can be used for the analysis of executable models, regardless of the modeling framework, and for making experimentally quantifiable predictions.

## Introduction

Biological systems are remarkable examples of complex dynamical systems. The dynamics of these systems involve extreme concurrency and interactions across multiple scales of biological organization. For example, the states of individual cells are determined by internal molecular processes governed by molecular interaction systems. These cellular states influence cell-cell interactions, which collectively give rise to macroscopic behavior, but the ensuing macroscopic state of the system, such as establishment of cellular structures (e.g., blood vessels) or nonhomogeneous distributions of diffusible molecules, also feeds back on the “lower” intracellular molecular systems and their states. The complexity of biological systems is further compounded by the fact that they are open and react to time-varying input received from their environment. A reactive system must respond to each stimulus as it occurs, often needing to respond to many stimuli concurrently [1], [2]. The structure of the system is also typically dynamic, with its components being repeatedly created and destroyed during the system’s lifespan, adding yet another level of complexity.

Over the last decade, systems biology research has yielded a plethora of data pertaining to biological systems. To facilitate further analysis and systems-level understanding, this information is often integrated into large scale models using a variety of mathematical and computational approaches. A general class of models are so-called executable models [3]. Such a model defines how, given certain events, the system transitions from one state to another. State-based formalisms can be used to construct computational models that describe the complex dynamics of reactive systems, including biological systems. Such models are typically highly nonlinear and nondeterministic, and can simulate very large systems [3]. There are a variety of model classes and approaches that can serve as the basis of executable models of biological systems, among which are Boolean networks, Petri nets, statecharts and process calculi [4]–[24].

Any given model class, be it a Boolean network or a system of stochastic differential equations, comes furnished with the appropriate framework and tools for analyzing system dynamics. For instance, to assess the sensitivity of a continuous dynamical system to small perturbations, we may compute the Lyapunov exponent based on Euclidean distance as a measure of state similarity [25]; but for a Boolean network, an appropriate distance metric may be the Hamming distance [26]. Even for a more esoteric model class, say a multilevel discrete dynamical system (of which a Boolean network would be a special case, with every element in the system restricted to being binary-valued), it is possible to come up with a reasonable distance metric that would quantify state similarity. Although each modeling formalism provides avenues for simulation and analysis of system dynamics, general “model independent” approaches for analysis of dynamical behavior are lacking.

The problem becomes greatly compounded once we start moving away from abstract models that entail significant assumptions and reductions of scale; for instance, a system of ordinary differential equations that models a genetic network assumes a well-mixed homogeneous system of cells (all assumed to be in the same state) to allow for molecular concentrations to be used in the model, since on a single cell level, such a model is not appropriate due to low molecular counts. Fine-grained models that incorporate and bridge molecular (both intracellular and diffusible extracellular) and cellular information present a significant challenge to the study of system dynamics. Although a state (“snapshot”) of the system can be defined, encompassing information such as spatially varying molecular concentrations, cell types and positions of cells, or activation status and other functional states of individual cells, it is far less clear how to study the dynamics of the system that incorporates all this information. Loosely speaking, if we consider the collective information embodied in a state of the system, then system dynamics amounts to information flow; the information in a given state is related to the information in a predecessor state.

Classical Shannon information theory is based on modeling the distribution of symbols that need to be fixed in advance [27]. Algorithmic information theory, on the other hand, allows the quantification of the information content of any object that can be represented on a computer, without prior assumptions of the symbols or their distributions [28]. Instead, the relevant subsequences and their relationships within the objects are determined on the fly by the data compression model. This makes algorithmic information theory a powerful foundation for model independent analysis of dynamics. The main practical benefit of this concept is that it can easily be applied to real life data using a computable approximation of the theoretical information distance called the normalized compression distance (NCD) [29]. We have previously applied the NCD to study the dynamical behavior of discrete network models [30]–[32]. The NCD based analysis corroborated and generalized results obtained with model-specific tools. Here, we show that the NCD based analysis of system dynamics can also be applied to general executable models.

**Figure 1:**
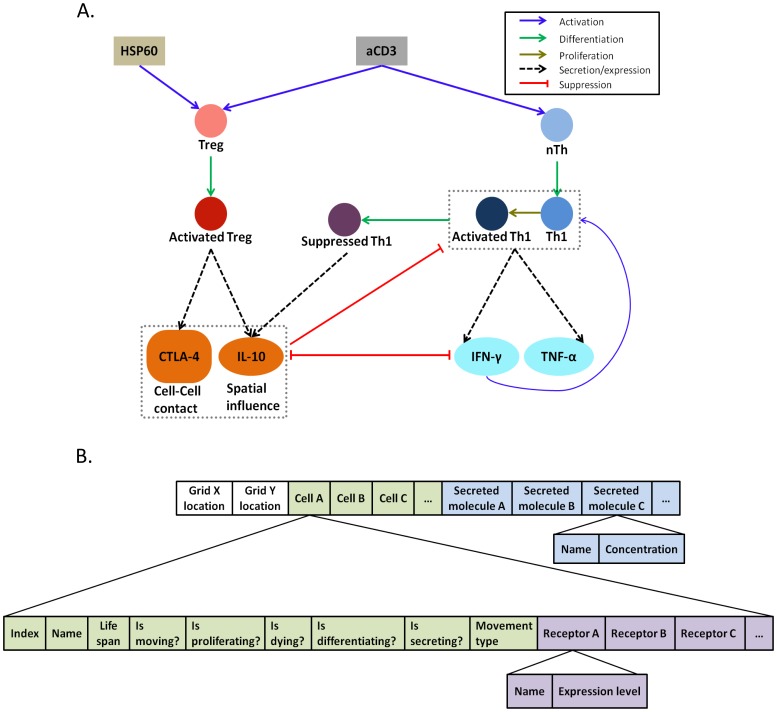
Representation of the simulated biological system. A) A cartoon diagram of the simplified biological system underlying the modeling. HSP60 and aCD3 serve as activators of the Treg and nTh populations by binding TLR2 and CD3 respectively. The activated Tregs feature two effector molecules that communicate inhibitory signals to other effector T cells, thereby suppressing proliferation and inflammatory cytokine secretion (IFN-γ and TNF-α). CTLA-4 is a membrane bound inhibitory signal that binds B7 molecules on effector T cells, thus requiring direct cell contact between Tregs and effector T cells. IL-10 is a secreted molecule that binds IL-10 receptor on effector T cells and thus has spatial influence on the population. IFN-γ acts as an activator of the effector T cells [42]–[47]. TNF-α secretion was measured, but its pro-inflammatory activity was not simulated in this simplified model. B) Structure of the state files. The files are tab delimited text files. There is a separate file for each time point of the model execution period. Each line in a file corresponds to a specific grid location, and contains the information of all the cells and molecules that are present on that grid location at the time point represented by that file. The first segment of information in the line shows the cells (see green segment in the figure). For each cell on that grid location there are its specific details: its index in the database, its name and remaining life span, and its behavioral state. In addition, for each cell there is a list of the receptors it expresses (purple segment). For each receptor, there are its specific details: its name and its expression level. The last information segment in the line is the diffused molecules information (blue segment). For each molecule there are its name and concentration on that specific grid location.

As a proof of principle, we apply the NCD based analysis on a statecharts based executable model of immune decision making and immune cell behavior [33], [34] (Figure 1a). The model describes in a simplified fashion the capacity of regulatory T cells (Tregs) to suppress inflammatory T cell effector functions and the dependence of this suppression on Cytotoxic T-Lymphocyte Antigen 4 (CTLA-4), Interleukin-10 (IL-10) and interferon-gamma (IFN-γ) levels (Figure 1a) [33], [34]. The model was generated using GemCell, a statecharts based generic modeling tool that facilitates detailed simulations of dynamics of multi-cellular biological systems [35]. Execution of models built using GemCell produce result files containing a detailed description of the state of the system at any given time point (Figure 1b). The modeling framework enabled us to run the model not only under so called “normal”, or wild type initial conditions, but also allowed us to generate predicted dynamics of the system under various experimental initial conditions, such as knock-outs and changes in the ratios of the different cell populations.

We present a proof-of-concept methodology for analyzing the dynamics of complex biological models by building on and generalizing a previously published methodology demonstrated on Boolean network models and gene expression data [30], [31]. We show that this extended approach can be successfully applied to complex models containing multiple types of interactions and dynamics. To demonstrate this approach, we apply it to a simplified example of a fine-grained molecular simulation model, in which there is a low-level representation of the molecular processes in addition to complex cellular level simulation. This approach can be easily applied as-is to more detailed and highly complex multiscale models and is, therefore, independent of the modeling framework.

## Methods

### The Simulation Framework

To show the feasibility of our methodology, we applied it on the output of an executable model that simulates the effect of heat shock protein (HSP60) on the interactions between two populations of T cells - Tregs and nTh cells, and the results of these interactions [33], [34]. This model was created using GemCell, an agent-based generic modeling platform [35]. GemCell has three components that continually interact during model execution: 1) A computational module created in statecharts [36], [37] that describes the generic rules of cellular behavior; 2) database of biological specifics (DBS) - a MySQL database that contains the biological data pertaining to the specific system being modeled, and 3) output in the form of animation and result files [35].

The database that contains the data for the model holds several types of information - the number and types of cells and molecules in the system, numerical discrete parameters, such as concentrations and affinity levels, and general rules such as which cells express which receptors, which molecules can potentially bind and interact, and the emergent cellular behavior of these interactions. The database is constructed in a way that the data in it can be easily changed, and the user can create different sets of initial parameters for the execution of the model, or different sets of interaction rules [35].

The spatial conformation of the model is a 20×20 2D grid. There are no limits on the number of cells that can be present on each grid location at any given time point, and there can also be several types of molecules with different concentrations on the same grid location. We started each execution with a total of 100 cells dispersed randomly across the grid.

The model is synchronous and simulates the changes in the dynamics of the system over 30 hours with time resolution of one hour. Thus, in our analysis 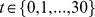. Model execution produces a set of detailed result files that contain information about the state of the system at each time point. An overview of the format of the result files and the information they contain is shown in Figure 1b. The state of the system is defined by these text files that include the number, type and state of cells and the number, type and concentration of molecules at each time point. The dynamical behavior of the model refers to changes over time of the entire state of the system.

We executed the model under several different initial conditions: wild type, knock-outs of three major molecular components - IL-10, IFN-γ and CTLA-4, and different ratios of cell quantities of the two initial cell populations. We also generated a random model, in which the numerical parameters we used were random, but the interaction rules remained the same as in the wild type model. For each initial condition we generated 50 runs 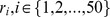 of the model to allow for statistical analysis. More details of the model structure are available in the supporting information (File S1).

### Analysis Tools

We used algorithmic information theory [28] to measure the distance between two states of the system. The normalized information distance (NID) [38] measures the amount of shared information between two objects. The normalized compression distance (NCD) approximates the NID between two objects, as the NID is not computable. A detailed development of the NCD as a valid approximation of the NID is provided in [29]. In our case, the two objects are text files that contain the information about the state of the system at two time points. The NCD is defined as: 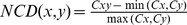, where 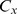 is the compressed size of the first object, 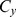 is the compressed size of the second object, and 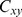 is the compressed size of the concatenation of the two objects (states). We used the LZMA2 compressor to compress the files that encode the state of the system. We refer the reader to File S1 for more details.

We used non-metric multidimensional scaling (MDS) to visualize the state trajectories in a low dimensional space. The non-metric MDS algorithm was initialized with the solution of the classical multidimensional scaling algorithm. We used Kruskal’s stress criterion as an objective function.

For MDS, we created a global NCD distance matrix between all the states from each of the “setups”, the wild type, and the IL-10, IFN-γ and CTLA-4 knock-outs. For any two setups, the NCD distance matrix has a size of (50×31, 50×31) = (1550,1550). For all the four setups, the NCD distance matrix has a size of (4×1550, 4×1550) = (6200×6200). The overall computational cost of building the NCD distance matrix is high, but can be mitigated by the fact that the computation can be done in parallel (see File S1).

To quantify the information flow within the system, we defined the convergence or divergence of states based on whether the distance between them increases or decreases over time. We measured the distance between two states with the NCD. First, we computed the NCD between two states 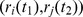, 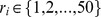, 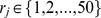 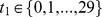, 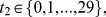: 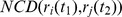. The NCD values were zero when *i = j* and *t_1_ = t_2_*. These values were eliminated from the analysis. Next, we computed the NCD between the consecutive states: 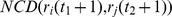
_._ This was repeated for all possible pairs 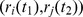. Due to computational constraints, we randomly selected twenty simulation runs and as a result, we obtained a map of convergence and divergence of distances over time. For this map, we computed the probability density function (pdf) of the NCD points using two dimensional Gaussian kernel density estimation with automatic bandwidth selection [39]. The grid size for the kernel density estimation was 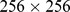.

For visualization, we chose contour levels for the estimated density such that we had high resolution in the regions of small probability values and low resolution in the regions of large probability values (see File S1).

## Results

**Figure 2:**
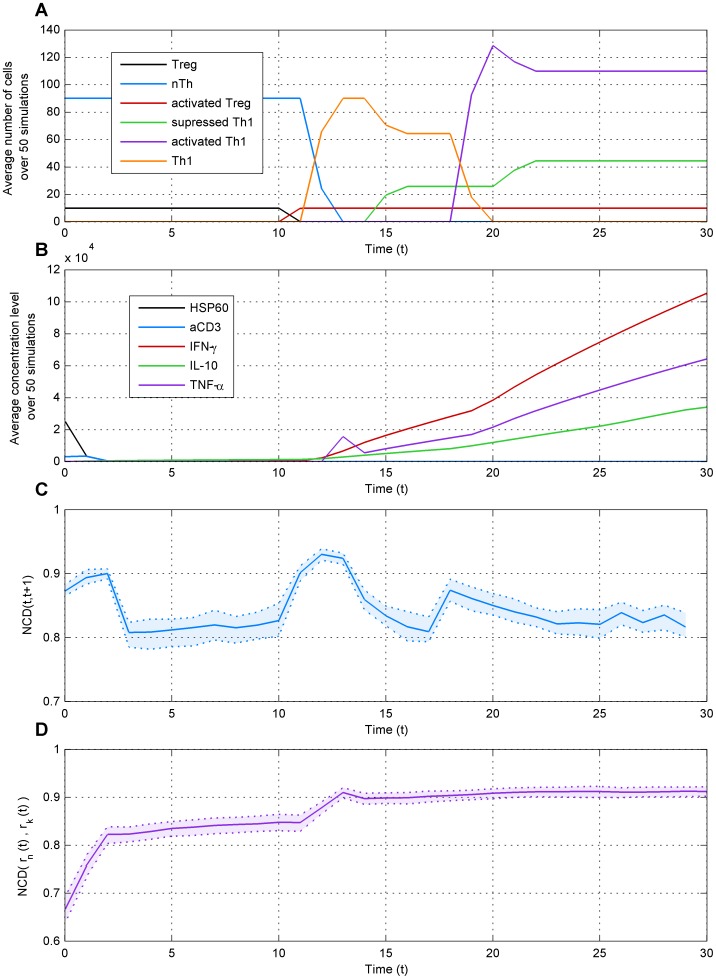
Dynamics of the wild type (WT) simulation. a) Changes in the number of cells in each cell population over time, averaged over 50 simulations and b) changes in the concentration levels of the secreted molecules over time, also averaged over 50 simulations. In c) NCD over time between two consecutive states of one simulation and d) changes over time in the NCD between the same state taken from two distinct simulations. The average of 50 simulations is shown with the 5th percentile confidence interval. The major changes in NCD in (c) co-occur with the events in a) and b). There is a clear trend of divergence in d) as the distances between trajectories from different runs increase over time.

The behavior of the simulated system under wild type conditions, in terms of the different cells and molecules present in the system, is shown in Figure 2a–b. These can be used to quantify what is happening in the simulation over time. A more detailed description of the model can be found in the supporting information (File S1) and in [33], [34]. The execution of the model under wild type conditions begins with two populations of cells – ten T regulatory cells (Tregs) and 90 naïve T cells (nTh) [40]. In addition, there are two secreted agents diffused in the environment (medium) – HSP60 and aCD3, which serve as activators of the two cell populations (Figure 1a). There is no additional secretion of these two agents, so, as execution time progresses, these molecules gradually disperse in the environment and interact with the cells, and, thus, progressively disappear from the system (Figure 2b). By time point 2, they both almost completely vanish from the model. The activation of the Treg population by HSP60 and aCD3 causes it to differentiate to a new population of cells – activated Tregs, and the activation of the nTh population by aCD3 leads to its differentiation to another new population of cells – Th1 Cells (Figure 1a). These two differentiation events occur around simulation time points 10–11 (Figure 2a). At around time point 12–13, the new populations of cells start secreting molecules that were not previously present in the environment– IL-10, IFN-γ and TNF-α (Figure 1a, Figure 2b). The interaction between the activated Tregs and the Th1 cells causes parts of the latter to go through another differentiation event at around time point 15, producing another new cell population – suppressed Th1 cells (Figure 1a, Figure 2a). This new population switches from secreting IFN-γ and TNF-α to secreting IL-10 (Figure 1a). At around time point 18–19, the Th1 population goes through a proliferation event towards a new cell population, activated Th1 cells (Figure 2a), which in turn secretes higher levels of IFN-γ and TNF-α (Figure 1a). The activated Th1 cells are also regulated by the activated Tregs, and go through a differentiation event that produces more suppressed Th1 cells at around time point 22 (Figure 2a).

To see if the NCD is able to detect these events from the state data, we first compared two consecutive states (Figure 2c). There is a major change in the NCD at three time instants. The first event *t* = 1 corresponds to the depletion of HSP60 and aCD3 molecules as discussed above. Consecutively, events at *t* = 11 and *t* = 17 correspond to two differentiation events. This indicates that relevant information about the system’s behavior can in fact be extracted with the NCD. The comparison of distances between two trajectories over time indicates a general diverging trend in the dynamical behavior, due to the random movement of the molecules in the system (Figure 2d). We present a more detailed analysis of the states of the system in Figure S1, where we analyze the cell populations and the secreted molecules separately. The results are consistent with what we have presented in Figure 2c–d.

To study the behavior of the system in more detail, we computed a distance matrix to measure the similarity of all the pairs of states (see Methods). We applied the non-metric multidimensional scaling algorithm to this distance matrix to project the distances into three-dimensional space. We studied the similarity of the trajectories given by different perturbations of the system, by connecting the resulting data points in temporal order. While a metric, the dynamic range of NCD is not linear. Thus, the transformation to a more general rank based space allows the objective comparison of distances in a scale independent manner.

**Figure 3:**
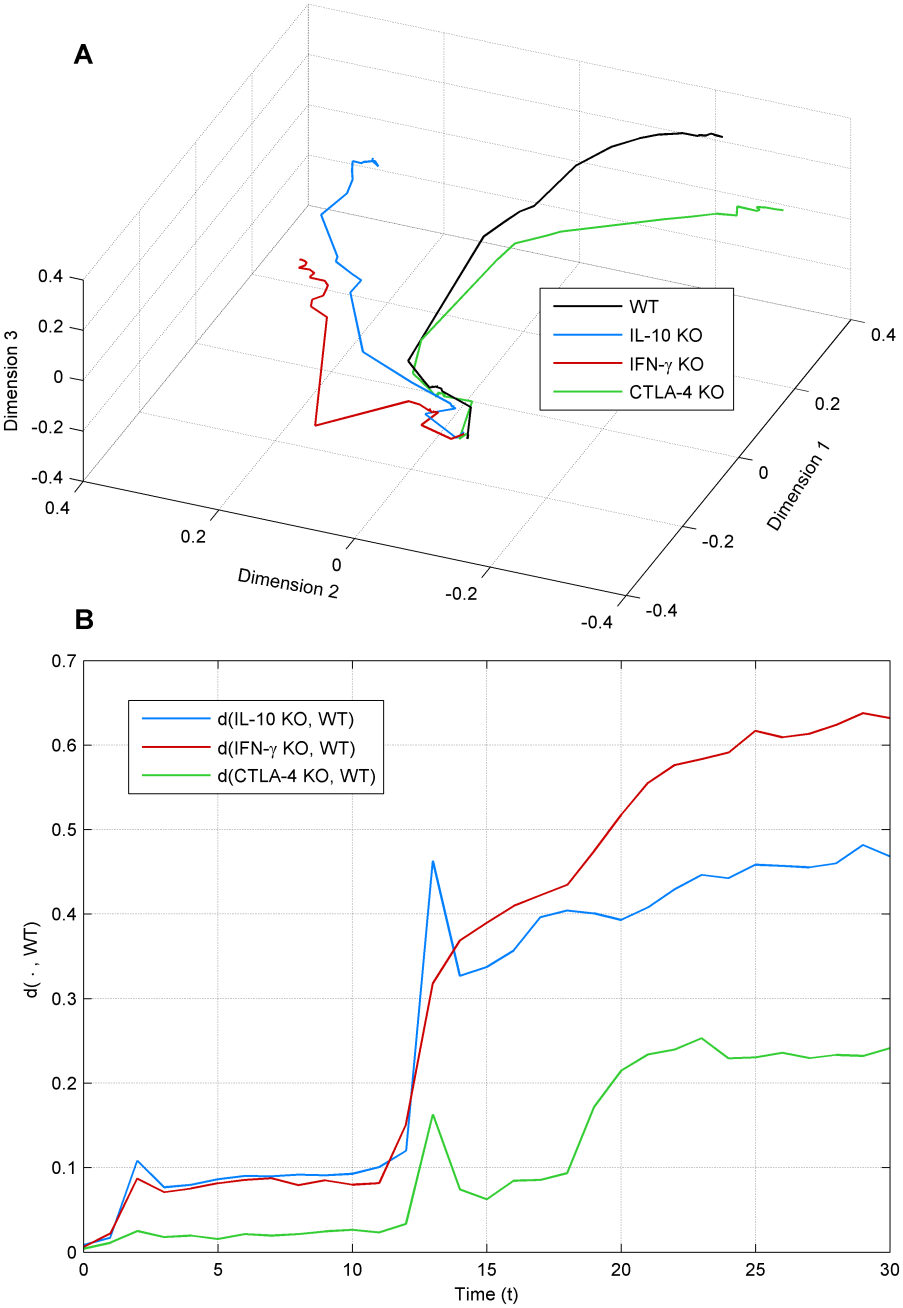
Non-metric multidimensional scaling of state trajectories. a) 3D state trajectories of wild type (WT), IL-10, IFN- γ and CTLA-4 knock-outs (KO); b) the Euclidean distance between the trajectories in relation to wild type, denoted as (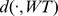). Average of 50 simulations is shown.

Figure 3 shows the trajectories for the wild type as well as for IL-10, IFN-γ and CTLA-4 knock-out systems. It is clear that there is a diverging trend of the trajectories. To quantify this divergence, we computed the pair-wise Euclidean distance between each of the trajectories of the perturbations and the wild type trajectory (within the multidimensional scaling solution). Here we can see that the effect of CTLA-4 knock-out is smallest as the trajectory is most similar to that of the wild type. IL-10 and IFN-γ knock-outs clearly cause more dramatic effects on the system’s dynamics in comparison to the wild type. We can also observe that the trajectories of IL-10 and CTLA-4 are similar to each other, while that of the IFN-γ is different. This corresponds to the similarities in the inhibitory effects of IL-10 and CTLA-4 on the cell populations, in contrast to the pro-inflammatory effect of IFN-γ. We present a similar analysis in Figure S2, where instead of knock-outs, we alter the ratios of the cell populations of Tregs and nTh at the beginning of the simulation.

**Figure 4:**
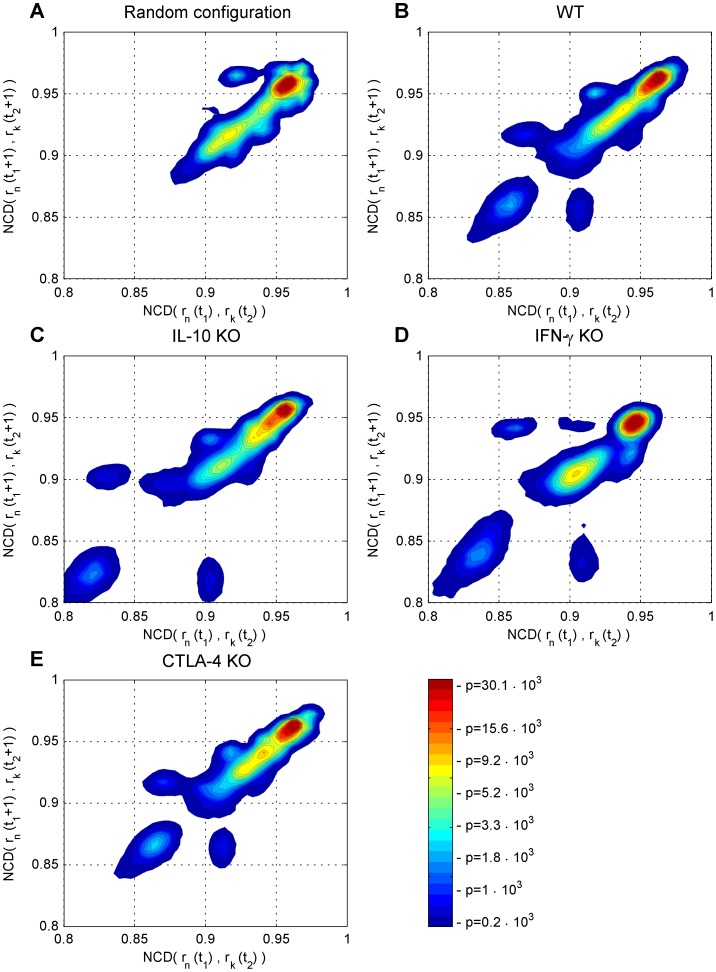
Quantification of the information flow in the random model, the wild type and perturbations. Information flow in a) random model, b) wild type (WT), c) IL-10, d) IFN-γ and e) CTLA-4 knock-outs (KO). While the information flow in the random model is focused in a narrow area, the wild type and knock-out simulations are more diverse, with each system exhibiting characteristic dynamical behaviors.

While the MDS analysis provides a clear insight into the system’s dynamics, it is based on a projection into a low dimensional Euclidean space. This representation contains only the most relevant information in terms of the minimized stress criteria. To obtain an alternative, more quantitative view into the dynamics, we study the information flow in the system over time (see Methods). The resulting maps of convergence or divergence of state trajectories are shown in Figure 4. Visually, it is clear that each experimental condition exhibits a high peak of the probability density function approximately in the same region of the NCD values, where most of the points lie. The differences between the systems arise in isolated areas (islands) of pair-wise distances between states. These events are much less frequent than the events at the highest peak of the density, but are still clearly established and consistent between different simulations.

In addition to the wild type and knock-out conditions, we also analyzed the behavior of a random model (see Methods), to see the effect of the constraints of the structure of the model on its dynamical behavior (Figure 4a). This random model shows very concentrated dynamical behavior as the distance between the initial and consecutive states remains approximately the same (most points lie on the diagonal). In addition, the shape of the distribution is quite similar to the main peak seen in the other simulations. In comparison, the wild type simulation shows a wider range of dynamical behavior, including islands where the information flow is constrained (Figure 4b). The conclusions provided by this analysis are consistent with those drawn from the multidimensional scaling study. Here as well, the CTLA-4 dynamical behavior (Figure 4e) is more similar to wild type than the other two perturbations, and CTLA-4 and IL-10 are more similar to each other than to IFN-γ. In terms of similarity to the wild type, CTLA-4 is followed by IL-10 and IFN-γ (Figures 4c–d).

**Figure 5:**
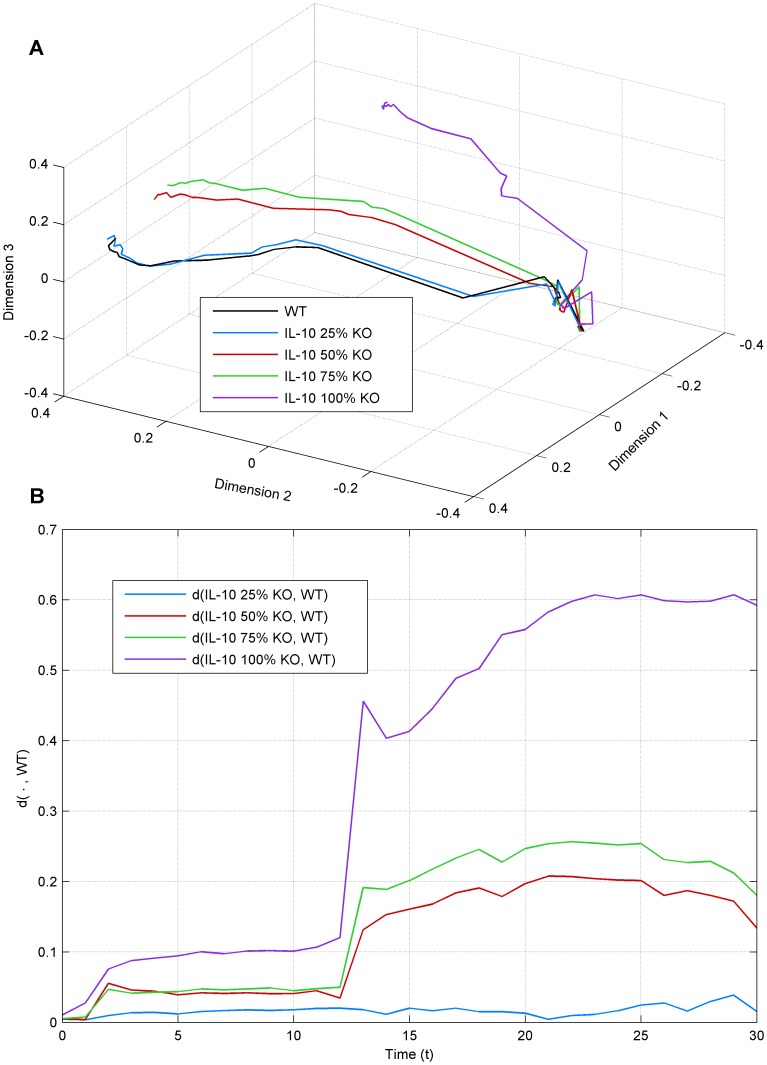
State trajectories for the IL-10 partial knock-outs (KO). Wild type IL-10 (WT) is compared with knock-outs at 25%, 50%, 75% and 100% efficiency. a) 3D representation of state trajectories. b) The Euclidean distance between wild type and knock-out trajectories is denoted as 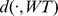). While the knock-outs are performed in linear steps, the effect on the dynamical behavior is nonlinear. 25% knock-out has only a small effect on dynamics while 50% and 75% show approximately equal effect, which is not as strong as with 100%. Data is shown as an average across 50 simulations.

To assess the degree to which such global information-theoretic analysis can reflect experimentally quantifiable phenomena, we performed an additional simulation. So far, we have considered perfect knock-outs of secreted molecules. In real biological systems the knock-out is hardly ever perfect, but only lowers the expression level of the target molecule to a certain degree. Such a partial knock-out can be quantified by measuring the expression of the molecule before and after the knock-out. Analogously, the effect of the knock-out could be observed from the representation of Figure 2b. However, this simple readout says nothing about the effect on the dynamical behavior. Our analysis can be used to understand the effect of knock-out efficiency on system dynamics. To prove the concept, we simulated a knock-out of IL-10 at 25, 50, 75, and 100% efficiency (Figure 5). Here we clearly see that 25% knock-out has very little effect on the dynamics when compared to the wild type. We, therefore, argue that higher efficiency is needed to have a biological effect. The 50% and 75% knock-outs clearly have a stronger effect on the dynamics, but they are still far from the perfect knock-out. Thus, the observed biological response would also be very different depending on the knock-out efficiency. The convergence-divergence maps for the partial knock-out experiments show that the dynamics become increasingly different with the knock-out efficiency (Figure S3). However, major effects between knock-outs cannot be visually observed from this analysis and thus the result presented in Figure 5 is more informative as a visualization for analyzing partial knock-outs.

## Discussion

Common practice in complex systems analysis is to analyze dynamical systems with model class specific tools. These are highly informative and effective in revealing the specific properties of the systems. However, as these methods are specially designed for each model class, the comparison and generalization of results between classes is challenging. In our previous work, we have successfully applied algorithmic information theory to Boolean and ternary network models [30], [31]. In this study, we are extending our previous approach to more fine-grained molecular models. This is the first approach that has been developed and successfully applied to the analysis of statecharts based systems. An algorithmic information theory based approach was able to extract relevant information from the highly complex state representation of the model, which incorporated both molecular and cellular information. The approach demonstrated here can be successfully applied to the output description of the state of the system of any executable model, independent of the modeling framework used. A comparable analysis with a Shannon information based approach would be difficult to apply in practice. In order to use a distribution based distance measure, the files would need to be encoded so that the alignment of the symbols is preserved. In addition, the state structure can also change, e.g. when the number of cells in the model increases due to cell division. In such cases, devising a classical information measure to compare the states before and after cell division requires an intimate knowledge of the significance of the different parts in the state description. Our NCD based approach was able to solve these problems automatically by using sequence complexity instead of symbol probabilities.

We used different data representation techniques that allowed us to observe the informational dynamics of a model of immune decision making and immune cell behavior. Multidimensional scaling based analysis allowed the representation of state trajectories in low dimensional space. Such representation will allow, for instance to study the relationships and transitions between attractors or steady states of the underlying system. With partial knock-outs we presented an example of how this approach can be used to produce experimentally quantifiable biological predictions in addition to general theoretical insights on the basis of system-level information dynamics.

We also studied the information flow in the systems. For this type of systems the sampling of the whole state space is not feasible and thus, we cannot build a complete map of the dynamical behavior. However, using a randomized model as a background and analyzing the range of observed dynamics in different knock-outs we were able to establish clear differences in dynamical behavior. We observed multiple domains where the information flow was constrained in comparison to random or wild type models. While we have shown that these observations can be informative about system dynamics, further development of systems theory is needed before the characteristics of such a limited sampling of state space can be tied to global dynamics of the system. Any computational model is a significant simplification of the real system. In this study, our model describes in a simplified fashion the capacity of regulatory T cells (Tregs) to suppress inflammatory T cell effector functions and it characterizes the dependence of this suppression on Cytotoxic T-Lymphocyte Antigen 4 (CTLA-4), Interleukin-10 (IL-10) and interferon-gamma (IFN- γ) levels. The goal of the model is to capture the interplay between these molecules, which subsequently leads to the control of cell proliferation and differentiation, analogously to the events in real biological systems. Our analysis is able to identify proliferation and differentiation events from the time series of output files that contain the state of the entire system. The timing of these events in the NCD analysis is consistent with what we expect from the simulation of the model. The further analysis where we compare the trajectories of the system in response to different knock-outs provides more in-depth knowledge on how the global behavior of the system is altered. Such system level properties cannot be quantified directly from the model output or from observing the biological system by any single experiment. Predictions derived from the system level analysis can directly be tested, such as the expression level needed to have an effective knock-out of the molecules.

In recent years there has been a growth in the demand and the corresponding development of multiscale models of biological systems. These models capture multiple abstraction levels such as genes, proteins, cells, tissues, organs and even whole organisms. The dynamics of the biological systems captured by these models are inherently highly complex. Studying the global dynamics of biological systems has the potential to generate insights regarding the general state of the system. For example, some aspect of the global dynamics of a certain system may correspond to a shift that system goes through from a healthy state towards a disease state. Predictive models could be used to explore possible parameters that cause the simulated system to shift towards such a dynamical state of disease. These parameters could then potentially be experimentally measured or even manipulated in the lab. Similarly, parameter changes could be identified to trigger the system to return to its normal healthy state. Even though the model we used as a case study in this paper is a somewhat simplistic example of a fine-grained simulation model, the information theoretic approach we present offers a method of understanding system dynamics and using it for optimization or control.

In conclusion, we have shown that algorithmic information theory provides a suitable framework for the analysis of fine grained-molecular simulation models and arbitrary model classes, making model comparison more straightforward. The methodology based on this framework is applied to a state description of the system, which is an output of a simulation of the model, and this is model framework independent and can be successfully applied to models at various abstraction levels. The analysis of the dynamics of an immune system model also demonstrated the potential of this approach to make experimentally quantifiable predictions. This approach can be easily applied as-is to more detailed and highly complex multiscale models. We believe that the proposed methodology is a step forward in understanding the dynamics of complex multiscale biological models that represent the behavior of the whole cell or even organism [41].

## Supporting Information

Figure S1 **Dynamics of the wild type (WT) simulation in terms of molecular and cellular features.** NCD over time between two consecutive states of one simulation using only a), b) molecular features and c), d) cellular features. In a),c) NCD over time between two consecutive states of one simulation and b),d) changes over time in the NCD between the same state taken from two distinct simulations. While molecular features are more consistent with the overall dynamics, both analyses, the one presented in figure 2 of the main paper and the present one, give consistent results. Average over 50 simulations and the 5 percentile confidence interval are shown.
(TIFF)

Figure S2
**Non-metric multidimensional scaling of state trajectories.** a) 3D state trajectories for simulations with different ratios of initial cell types: 10–90 (wild type (WT)), 30_70 ratio, 50_50 ratio, 70_30 ratio, and 90_10 ratio; b) the Euclidean distance between the trajectories in relation to wild type, denoted as 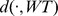. Average of 50 simulations is shown. Each ratio system leads to distinct state trajectories and thus the results are analogous to different knock-out simulations.(TIFF)

Figure S3 **Quantification of the information flow in the partial knock-outs (KO) of the IL-10 perturbation.** Information flow in the a) wild type IL-10 (WT), b) IL-10 KO at 25% efficiency, c) IL-10 KO at 50% efficiency, d) IL-10 KO at 75% efficiency e) IL-10 KO at 100% efficiency. The partial knock-out experiments show that the dynamics become increasingly different with the knock-out efficiency.
(TIFF)

File S1 **Supporting information.** Additional information regarding analysis methodologies and model description.
(DOC)

File S2 **Knockout simulations.** Data files used for knockout simulation analysis.
(ZIP)

File S3 **Cell population ratio simulations.** Data files used for cell population ratio analysis.
(ZIP)

File S4 **Random model.** Data files used to generate a random model.
(ZIP)

File S5 **Partial knockout simulations.** Data files used for partial knockout simulation analysis.
(ZIP)

File S6 **Cellular features.** Data files used to analyze only the cellular features.
(ZIP)

File S7 **Molecular features.** Data files used to analyze only the molecular features.
(ZIP)

File S8 **Cellular and molecular data from the model.** Data files with information from the model used to generate Figure 2.
(ZIP)
