# Supplementary figures and images for: Information-Theoretic Analysis of the Dynamics of an Executable Biological Model
Source: PLoS One. 2013 Mar 19;8(3):e59303. doi: 10.1371/journal.pone.0059303 (PMC3602105; doi:10.1371/journal.pone.0059303)

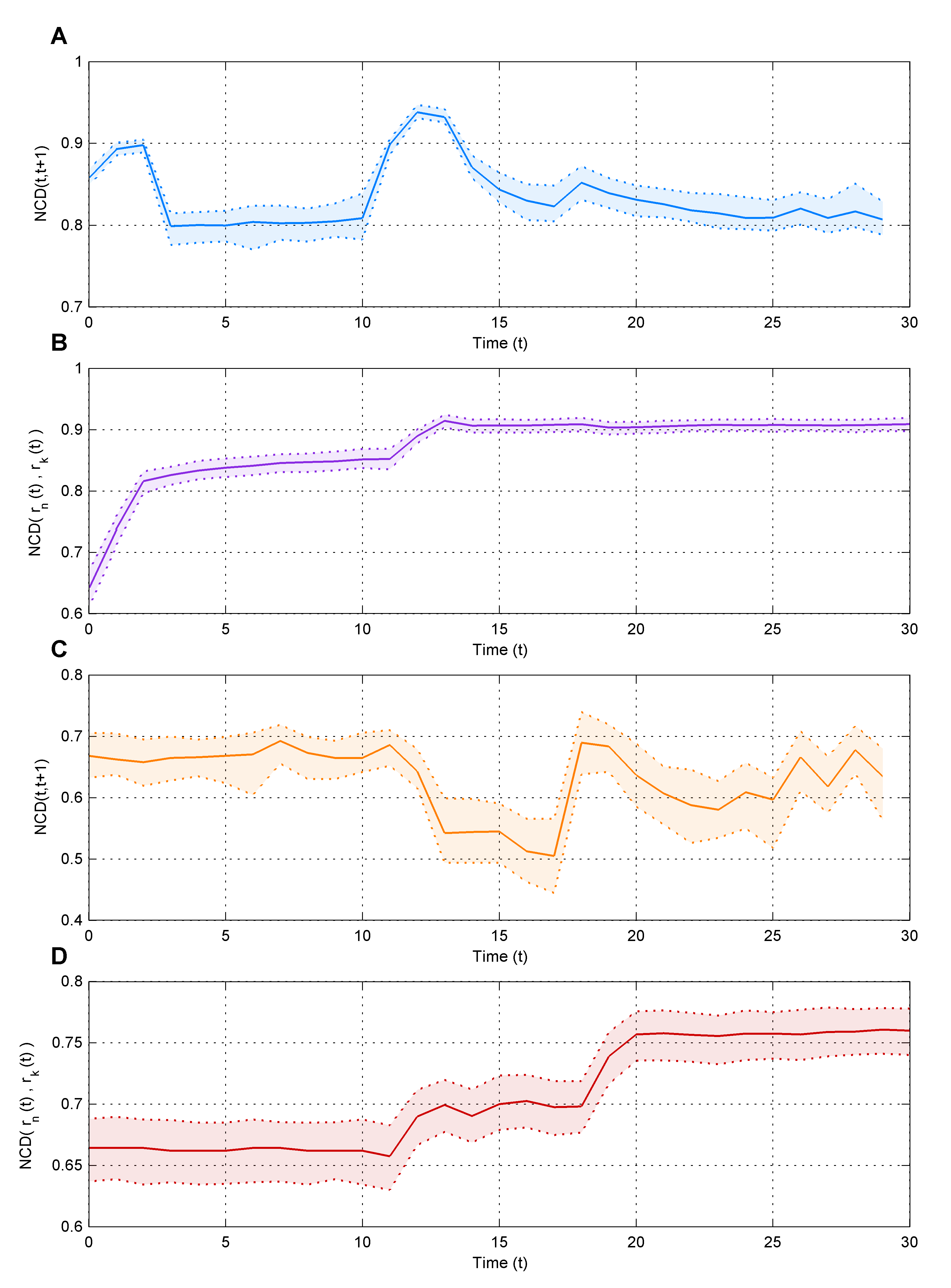

Supplement: Figure S1 — Dynamics of the wild type (WT) simulation in terms of molecular and cellular features. NCD over time between two consecutive states of one simulation using only a), b) molecular features and c), d) cellular features. In a),c) NCD over time between two consecutive states of one simulation and b),d) changes over time in the NCD between the same state taken from two distinct simulations. While molecular features are more consistent with the overall dynamics, both analyses, the one presented in figure 2 of the main paper and the present one, give consistent results. Average over 50 simulations and the 5 percentile confidence interval are shown. (TIFF) [file pone.0059303.s001.tiff]

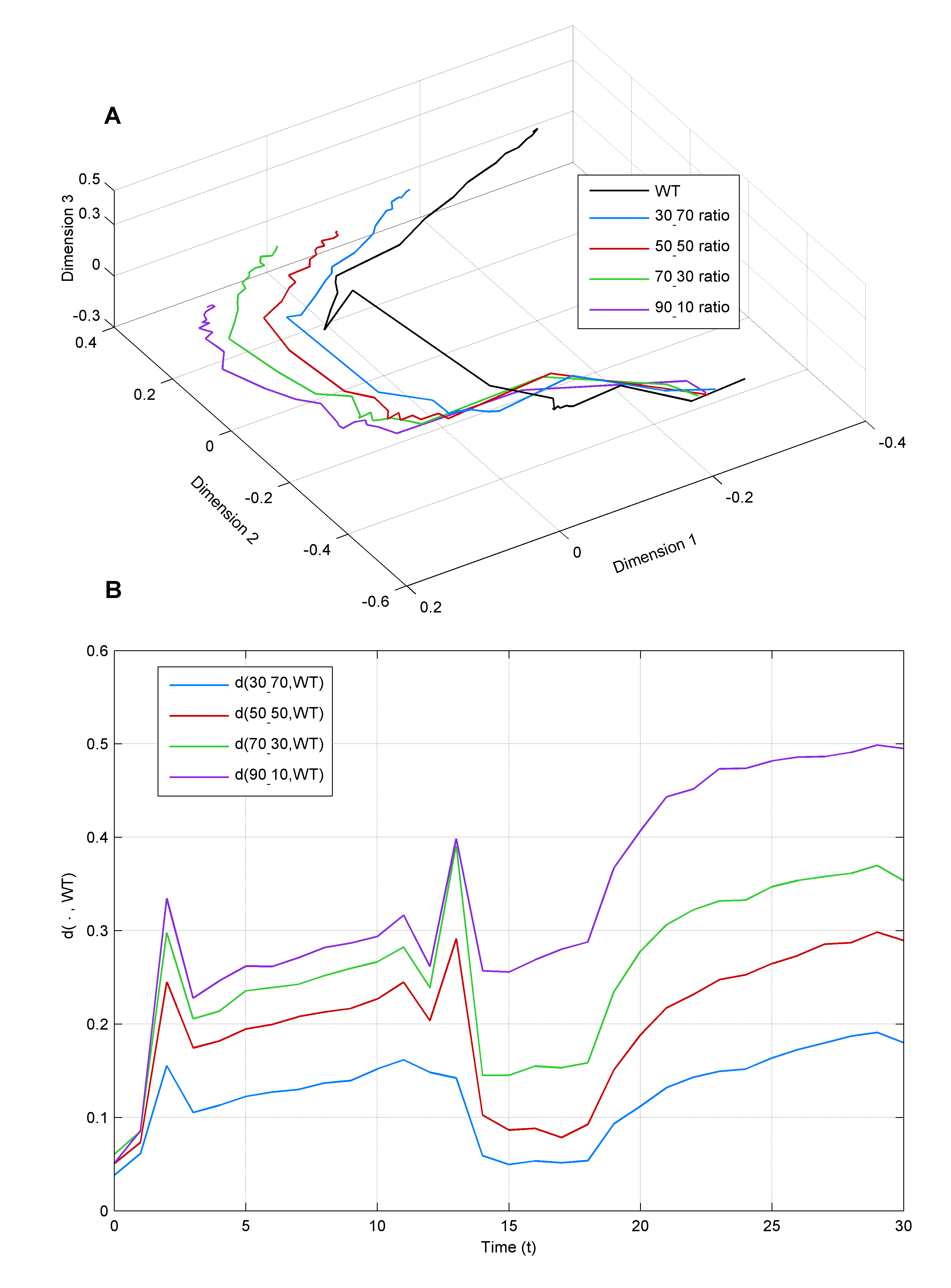

Supplement: Figure S2 — Non-metric multidimensional scaling of state trajectories. a) 3D state trajectories for simulations with different ratios of initial cell types: 10–90 (wild type (WT)), 30_70 ratio, 50_50 ratio, 70_30 ratio, and 90_10 ratio; b) the Euclidean distance between the trajectories in relation to wild type, denoted as . Average of 50 simulations is shown. Each ratio system leads to distinct state trajectories and thus the results are analogous to different knock-out simulations. (TIFF) [file pone.0059303.s002.tiff]

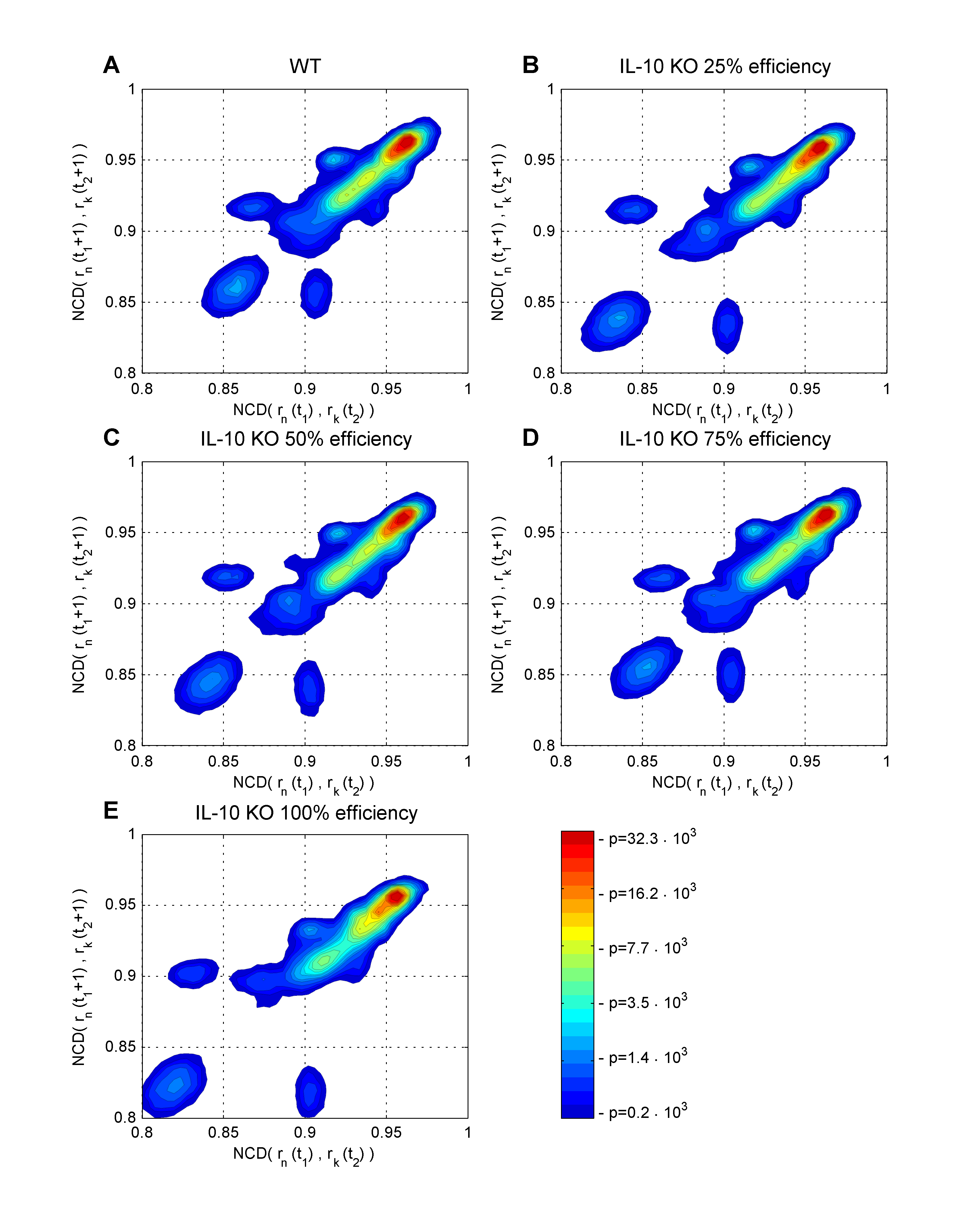

Supplement: Figure S3 — Quantification of the information flow in the partial knock-outs (KO) of the IL-10 perturbation. Information flow in the a) wild type IL-10 (WT), b) IL-10 KO at 25% efficiency, c) IL-10 KO at 50% efficiency, d) IL-10 KO at 75% efficiency e) IL-10 KO at 100% efficiency. The partial knock-out experiments show that the dynamics become increasingly different with the knock-out efficiency. (TIFF) [file pone.0059303.s003.tiff]
