# Supplementary material for: Information-Theoretic Analysis of the Dynamics of an Executable Biological Model
Source: PLoS One. 2013 Mar 19;8(3):e59303. doi: 10.1371/journal.pone.0059303 (PMC3602105; doi:10.1371/journal.pone.0059303)
Supplement: File S1 — Supporting information. Additional information regarding analysis methodologies and model description. (DOC) [file pone.0059303.s004.doc]

**Supporting Information**

**Data representation**

The encoding of the data in the text file affects the results of the compression of that file. We arranged the data in the files according to the location on the grid. Each line represents an (X, Y) grid location, and contains the data of all cells and molecules located on that specific grid location. The data includes names, the behavioral state for the cells, and the concentration/expression level for the molecules (see Figure 1b). We encoded the data in the files to one letter characters rather than strings (for names of cells and molecules), integers rather than floating numbers (for concentrations and expression levels), and Booleans (for cellular behavioral states). We then re-ordered the data in each line in ascending alphabetical order for characters and then ascending numerical order for integers and Booleans. This was done to avoid the effect of the variance of the order of the data recorded in the files due to probability in the model.

**NCD Estimation**

We use the real-life compressor xz Utils (http://tukaani.org/xz/) to compute the normalized compression distance (NCD) between two states of the system. The compression technique is based on the Lempel-Ziv Markov chain algorithm (LZMA2). Out of all the available compression algorithms (gzip, bzip2, LZMA implemented in the 7zip package etc.), xz Utils provides the NCD range that is the closest to the ideal interval of [0 1]. There are, however, challenges in working with real-life compressors. The NCD between identical files is greater than zero and between totally random files is less than one. This can be compensated by normalizing the NCD values in the [0 1] interval. For the data representation used in this work we observe NCD distances in the interval [0.64 0.94]. The MDS algorithm requires a symmetric distance matrix, as the NCD is not symmetric, we take the arithmetic mean of the two NCD values: NCD(file_i_, file_j_) and NCD(file_j_, file_i_), where the indices i and j indicate two generic files.

**MDS based data representation**

We transform the high dimensional state information into a three-dimensional representation using non-metric MDS, implemented in Matlab. We use the following parameter values: the goodness-of-fit criterion to minimize is Kruskal’s normalized stress1 criterion, the initial configuration of the Cartesian points is determined using the classical multidimensional scaling algorithm, the termination tolerance for the stress criterion and for its gradient is $10^{-4}$. The maximum number of iterations is set to 500. If the termination tolerance is not achieved in 500 steps, then the algorithm did not converge and we have a suboptimal configuration of points. The global dissimilarity matrix which is the input to the *mdscale.m* function contains $Ns \times Ns$ block matrices of dissimilarities between each combination of two experimental conditions out of the total of $Ns$ systems compared with the MDS algorithm. An element of the global dissimilarity matrix is $NCD(system_{i}(r_{n}(t_{1})), system_{j}(r_{k}(t_{2})))$, where $i\in\left\{\text{1,...},N_{s}\right\}$, $j\in\left\{\text{1,...},N_{s}\right\}$, $r_{n}\in\left\{\text{1,...},50\right\}$, $r_{k}\in\left\{\text{1,...},50\right\}$, $t_{1}\in\left\{\text{0,1,...},30\right\}$, and $t_{2}\in\left\{\text{0,1,...},30\right\}$. For each pair of systems, the NCD is computed between each combination of runs and time points. The NCD values are normalized to be in the interval [0 1]. The elements on the diagonal of the global dissimilarity matrix are set to zero.

**Contour levels**

In order to have clearly spaced contour levels, high resolution in the region of low probability and low resolution in the region of high probability, we need to display between 20 and 30 contour levels. We start with 201 equally spaced contour levels to have high resolution throughout the figure. The large number of contours offers a wide range of values from which to choose the optimal unevenly spaced levels. These levels are the same for the $Ns$systems that are compared. The selection algorithm is summarized in the following pseudo code:

1. For each system, select between 20 and 30 unevenly spaced contour levels from the 201 equally spaced contour levels:
   1. Compute the area ratio between the area of the candidate contour and that of the last selected contour
   2. Select the candidate contour if the area ratio is above the given threshold
2. Merge into one list the unevenly spaced contour levels of all the systems
3. Reduce the number of levels from the combined list:
   1. Compute the relative difference between the actual value of the candidate level and that of the last selected contour
   2. Select the candidate contour if the relative difference is above the given threshold

The color palette illustrating the space between two consecutive contours starts from dark blue to dark red. The blue colors are mapped to low values of the contour levels and the red colors are mapped to high values of the contour levels. The transition of colors from dark blue to dark red is done in increasing order of the levels.

**Computational complexity of the NCD analysis**

Computing the NCD between two state representation files takes on average 1.95 seconds of real time in Matlab, on a computer running Windows 7 Professional, with processor Intel(R) Core(TM) 2 Duo CPU E8500 at 3.17 GHz, usable memory 3.21 GB and 32-bit operating system. If these computations were performed on a single computer as the one described above, the NCD analysis between two experimental conditions using 50 simulations for each condition and each simulation having 31 time points, would take approximately: 50 x 31 x 50 x 31 x 1.95 = 4684875 seconds (>54 days) of real time. The NCD computations are independent of each other and thus can be run in parallel. We used grid computing to run multiple NCD algorithms at the same time to considerably reduce the real time of the NCD matrix computation. Using the grid of 200 computers at the Tampere University of Technology, the NCD computation between two experimental conditions took between 6 and 10 hours, depending on the load of the grid.

## Model description: HSP60 regulates T-cell behavior

The model used in the analysis presented in this paper was created using GemCell, which a generic computational modeling framework described in . Here we describe the modeled biological system, the parameter calibration and some of the *in-silico* experiments performed using the model (adapted from , http://www.cs.tut.fi/sgn/csb/execmodel/).

## The biological system

In addition to serving as a chaperone, Heat shock protein 60 (HSP60) also functions as a molecular signal for the immune system: HSP60 is expressed in cells exposed to stress or immune activation, and has been found as a target of autoantibodies and autoimmune T cells in healthy individuals and in those suffering from autoimmune diseases . HSP60’s expression is up-regulated in inflammation, and HSP60-reactive T-cells have been observed in a large variety of inflammatory diseases . Accordingly, HSP60 has been considered a pro-inflammatory "danger signal". Nevertheless, HSP60 treatment has been demonstrated to arrest inflammatory damage. It was recently reported that administration of HSP60 led to the arrest of autoimmune disorders . These beneficial effects of HSP60 were marked by a shift in the autoimmune response from a Th1 phenotype to a Th2 phenotype . The researchers in proposed that the resolution of inflammation by HSP60 probably involves at least 2 pathways: (i) inhibition of T-cell chemotaxis into inflamed sites, and (ii) a switch towards Th-2 cytokine secretion .

A partial answer to the dominance of HSP60 as a target of autoimmune attack was provided by the discovery that HSP60 could directly activate APCs, including macrophages and dendritic cells, probably through binding to cell-surface receptors such as TLRs . It was recently shown that T-cells directly respond to HSP60 via the innate receptor TLR2 .

CD4^+^ CD25^+^ regulatory T-cells (Tregs) were shown to prevent autoimmune disease in mice , and they are also present in the blood of healthy humans . Tregs do not proliferate or produce cytokines upon polyclonal stimuli, and they are able to suppress T-cell proliferation and cytokine secretion such as IFN-γ and TNF-α . A recent study showed that Tregs are the target population of HSP60 by way of an innate signaling pathway that involves TLR2, and that HSP60-treated Tregs are markedly more effective than untreated Tregs in suppression of the proliferation of effector T-cells . Indeed, Tregs removal from the system completely prevents the inhibition of IFN-γ and TNF-α secretion by HSP60. This study found that HSP60-induced enhancement of Treg function involves both contact dependant and cytokine dependant mechanisms. HSP60 treatment enhanced the contact dependant (CTLA4) regulatory activity of Tregs and induced cytokine dependant (IL-10, TGF-β) regulation. Blocking of CTLA4, IL-10 or TGF-β partially but significantly inhibits the augmenting effects of HSP60 . These results are consistent with the finding that HSP60 treatment markedly augments IL-10 and TGF-β secretion in Tregs and probably also from their target T-cells.

## Model execution

#### Model Calibration; repeating lab results

Before we could perform *in-silico* experiments on our model, we had to calibrate it so it will reproduce the results measured in the “wet” laboratory experiments. If we can achieve these results, we can consider the data inserted into the model as verified. Once the data is verified, we can use the model to carry out *in-silico* experiments based on this data.

We followed lab experiments that tested the effect of HSP60 on T cell regulation in peripheral blood of healthy human donors . Experimental conditions were as follow:

“2 hours-HSP60-treated or untreated CD4+CD25+ T cells (Tregs) were co-cultured (1:10) with untreated CD4+CD25– T cells on mitogenic anti-CD3 Abs for 24 hours, and the cytokine levels were measured in the culture media.”

Four experiments were preformed:

- -HSP60 – No HSP60 was added to the medium of CD4+CD25+ T cells.
- +HSP60 – HSP60 was added to the medium of CD4+CD25+ T cells.
- IL-10 knockout – Anti-IL-10 Abs were added to the medium to inhibit IL-10 effect. This medium also contained HSP60.
- CTLA4 knockout – Anti-CTLA4 Abs were added to the medium to inhibit CTLA4 effect. This medium also contained HSP60.

Our model was able to repeat the results achieved in the lab. In laboratory and model experiments, the amounts of the indicated cytokines in the medium after 24 hours were similar:

- -HSP60 – Tregs were not activated and did not affect T cell activation by anti-CD3 Abs. The level of the Th1 cytokines – IFN-γ and TNF-α was high, and accordingly, the level of the regulatory cytokine IL-10, was low.
- +HSP60 – HSP60 led to the activation of Treg cells, which in turn down regulated Th activated cells. The secretion of IFN-γ and TNF-α was reduced by approximately 50%, and the secretion of IL-10 was tripled.
- IL-10 knockout – The absence of IL-10 led to a reduction in the ability of activated Treg cells to down-regulate activated Th cells. Consequently, although HSP60 was present, IFN-γ and TNF-α down-regulation was suppressed by approximately 25% and IL-10 elevation was suppressed by around 25%.
- CTLA4 knockout – Similar to the IL-10 knockout experiment, the absence of CTLA4 led to a reduction in the ability of activated Treg cells to down-regulate activated Th cells. Consequently, although HSP60 was present, IFN-γ and TNF-α down-regulation was suppressed by approximately 25%. The *in-silico* experiment suggests that IL-10 elevation, under these conditions, will be suppressed by around 25%. However, such measurements were not performed in the laboratory, and therefore this result is to be tested to confirm the model’s prediction.

Having said that, the calibration of the model is not perfect. The effect of the absence of IL-10 or CTLA4 on IFN-γ and TNF-α secretion was similar to that measured in the lab, but diverse. This difference in the outcomes can be a result of the reduction of the data, for example the absence of TGF-β, which might affect the fine tuning of the model’s experiments. Nevertheless, this diversion is within the borders of standard deviation, and therefore might simply points to variations within both biological and computational experiments.

#### In-silico experiments

Once our model was calibrated, we used it to carry out *in-silico* experiments. These experiments had two goals. The first was to reproduce previously done lab experiments and to learn about unmeasured experimental parameters. The second goal was to change experimental conditions and perform new biological experiments based on the calibrated, verified data.

We chose six parameters that were not or could not be tested in the “wet” lab, and used the four previously described experiments to evaluate them.

1. **Cytokine secretion pattern**

Using the computational model we were able to follow the cytokine secretion pattern over time. It is much harder to detect such delicate changes in cytokine levels in “wet” laboratory experiments. The amounts of secreted cytokines grow rapidly with time, and this elevation is controlled by the experiment conditions, as described earlier.

1. **Change in the number of Cells**

We measured the changes in the number of cells over time. In all experiments nTh cells were activated by anti-CD3 Abs after around 12-14 hours. During the following hours, some of these activated cells were down-regulated by the activated Treg cells while others continued to adopt the Th1 phenotype. Th1 activated cells proliferated once within the 24 hours measured, at about 18-20 hours.

-HSP60 – Most of the nTh cells were activated by anti-CD3 Abs and proliferated accordingly.

+HSP60 – The nTh cells were activated, but most of them were down-regulated by Treg cells both during activation and after Th1 proliferation. After 24 hours there were slightly more down-regulated cells than activated Th1 cells.

IL-10 knockout – the absence of IL-10 led to a late down-regulative effect of Tregs on Th1 cells. The reason is that the down-regulation is carried out only by CTLA4 which is expressed only at a later time point by most cells.

CTLA4 knockout – the absence of CTLA4 led to an earlier down-regulative effect of Tregs on Th1 cells, caused by an early secretion of IL-10 from Tregs in the process of activation. The latter synergetic effect of IL-10 and CTLA4 is eliminated. Therefore, although there are sufficient levels of IL-10 in the system, they are not enough to overcome the presence of IFN-γ.

1. **Chemotaxis**

To facilitate T-cell chemotaxis in our model, we added a symbolic chemotaxis agent to the environment, and an expression of a corresponding symbolic receptor on the T-cells.

-HSP60 – No chemotaxis.

+HSP60 - The addition of chemotaxis to the system caused an increase in the down-regulative effect of the Tregs.

IL-10 knockout - The introduction of chemotaxis into the system elevated CTLA4 regulation, since it increased the chances of contact between Th cells and Tregs.

CTLA4 knockout – the chemotaxis had no significant effect.

1. **Density**

To test the influence of a reduction in the density of the cells on their behavior, we increased our grid from a 12x12 grid to a 20x20 one.

-HSP60 – A minor elevation in the number of down-regulated Th1 cells. This is a result of a larger scatter of the basal secreted IL-10 from non-activated Tregs.

+ HSP60 - The expansion of the grid caused a reduction in the area of influence of both IL-10 and CTLA4, which in turn eliminated their late synergetic effect. Therefore the low density +HSP60 runs showed a marked difference in the Th regulation by Tregs, and lower levels of IL-10, and a corresponding higher levels of IFN-γ and TNF-α secreted by activated Th1 cells.

IL-10 knockout - As the effect of CTLA4 requires cell-cell contact, the increased distance between the cells on the larger grid caused the reduction in Th down-regulation. Even though we could observe some down-regulation of Th1 proliferation, the majority of the cells remained activated. Higher levels of IFN-γ were secreted by activated Th1 cells.

CTLA4 knockout - The CTLA4 knockout experiments with a larger grid show that the effect of IL-10 was reduced in about 10%. This was caused by a decrease in IL-10 concentration around Tregs, as a result of the expansion of the environment and the consequent reduction of the IL-10 accumulation in the microenvironments around the cells.

1. **Treg location**

To test the effect of the location of Tregs in our model, we changed the pre-run condition so that all Tregs will be located in the most left 10% locations of the grid, instead of randomly dispersed throughout the grid.

-HSP60 - The -HSP60 runs show an increase in the down-regulation of Th cells. This is a result of the massive IL-10 secretion in the relatively small area in which all Tregs were located. Since Th cells were spread randomly, Th cells that were located closer to the Treg area were affected by the high IL-10 concentrations. This IL-10 effect is more significant at the early stages of the run, before a large amount of IFN-γ is secreted by the activated Th1 cells.

+HSP60 - The addition of HSP60 to the system caused a reduction of more than 10% in IFN-γ secretion and a similar elevation of IL-10 secretion. This means that the high levels of IL-10 in the Treg zone already down-regulated most of the neighboring Th cells.

IL-10 knockout – the Treg zone limitations caused an elevation in cell-cell contact, and as a result the accumulative CTLA4 signal was able to almost overcome the IFN-γ signal.

CTLA4 knockout – the overall effect of IL-10 is the same as if the Tregs were spread randomly due to a very high IL-10 levels in a restricted zone.

1. **Treg to nTh ratio**

To test the effect of the ratio between the amount of Treg cells and the amount of nTh cells in the system, we increased the number of Tregs to be 20% of the total number of cells instead of 10%. The increase in the amount of Tregs elevated the down-regulative effect.

We could observe dramatic changes due to the density of the Tregs in the system and the consequent increase in both the size of the IL-10 microenvironment and CTLA4 signaling. Indeed, more Tregs secrete more IL-10, which is effective in the early stages of the simulation. Down-regulated Th cells will add to this effect by secreting more IL-10. Consequently there is a large decrease in the Th1 cytokine profile. Note that although the cell count of Th-reg cells is similar to the normal experiment, in fact the Th population number is reduced dramatically and therefore the down-regulative effect is much higher.

## References

1. Amir-Kroll H, Sadot A, Cohen IR, Harel D (2008) GemCell: A generic Platform for Modeling Multi-Cellular Biological Systems. Theoretical Computer Science 391: 276-290.

2. Sadot A (2008) On Specific and Generic Modeling of Complex Biological Systems. Rehovot: The Weizmann Institute for Science. 77 p.

3. Wallin RP, Lundqvist A, More SH, von Bonin A, Kiessling R, et al. (2002) Heat-shock proteins as activators of the innate immune system. Trends Immunol 23: 130-135.

4. Raz I, Elias D, Avron A, Tamir M, Metzger M, et al. (2001) Beta-cell function in new-onset type 1 diabetes and immunomodulation with a heat-shock protein peptide (DiaPep277): a randomised, double-blind, phase II trial. Lancet 358: 1749-1753.

5. Mor F, Cohen IR (1992) T cells in the lesion of experimental autoimmune encephalomyelitis. Enrichment for reactivities to myelin basic protein and to heat shock proteins. J Clin Invest 90: 2447-2455.

6. Cohen IR (1999) Tending Adam's Grden. San Diego, CA: Academic Press.

7. Hartl FU, Hayer-Hartl M (2002) Molecular chaperones in the cytosol: from nascent chain to folded protein. Science 295: 1852-1858.

8. Cohen IR (1991) Autoimmunity to chaperonins in the pathogenesis of arthritis and diabetes. Annu Rev Immunol 9: 567-589.

9. van Eden W, Thole JE, van der Zee R, Noordzij A, van Embden JD, et al. (1988) Cloning of the mycobacterial epitope recognized by T lymphocytes in adjuvant arthritis. Nature 331: 171-173.

10. Elias D, Markovits D, Reshef T, van der Zee R, Cohen IR (1990) Induction and therapy of autoimmune diabetes in the non-obese diabetic (NOD/Lt) mouse by a 65-kDa heat shock protein. Proc Natl Acad Sci U S A 87: 1576-1580.

11. Wick G (2000) Atherosclerosis--an autoimmune disease due to an immune reaction against heat-shock protein 60. Herz 25: 87-90.

12. Direskeneli H, Eksioglu-Demiralp E, Yavuz S, Ergun T, Shinnick T, et al. (2000) T cell responses to 60/65 kDa heat shock protein derived peptides in Turkish patients with Behcet's disease. J Rheumatol 27: 708-713.

13. Yokota SI, Hirata D, Minota S, Higashiyama T, Kurimoto M, et al. (2000) Autoantibodies against chaperonin CCT in human sera with rheumatic autoimmune diseases: comparison with antibodies against other Hsp60 family proteins. Cell Stress Chaperones 5: 337-346.

14. Hu W, Hasan A, Wilson A, Stanford MR, Li-Yang Y, et al. (1998) Experimental mucosal induction of uveitis with the 60-kDa heat shock protein-derived peptide 336-351. Eur J Immunol 28: 2444-2455.

15. Anderton SM, van der Zee R, Prakken B, Noordzij A, van Eden W (1995) Activation of T cells recognizing self 60-kD heat shock protein can protect against experimental arthritis. J Exp Med 181: 943-952.

16. van Eden W, van der Zee R, Prakken B (2005) Heat-shock proteins induce T-cell regulation of chronic inflammation. Nat Rev Immunol 5: 318-330.

17. Quintana FJ, Carmi P, Mor F, Cohen IR (2002) Inhibition of adjuvant arthritis by a DNA vaccine encoding human heat shock protein 60. J Immunol 169: 3422-3428.

18. Bockova J, Elias D, Cohen IR (1997) Treatment of NOD diabetes with a novel peptide of the hsp60 molecule induces Th2-type antibodies. J Autoimmun 10: 323-329.

19. Elias D, Reshef T, Birk OS, van der Zee R, Walker MD, et al. (1991) Vaccination against autoimmune mouse diabetes with a T-cell epitope of the human 65-kDa heat shock protein. Proc Natl Acad Sci U S A 88: 3088-3091.

20. Elias D, Cohen IR (1996) The hsp60 peptide p277 arrests the autoimmune diabetes induced by the toxin streptozotocin. Diabetes 45: 1168-1172.

21. Quintana FJ, Carmi P, Cohen IR (2002) DNA vaccination with heat shock protein 60 inhibits cyclophosphamide-accelerated diabetes. J Immunol 169: 6030-6035.

22. Elias D, Meilin A, Ablamunits V, Birk OS, Carmi P, et al. (1997) Hsp60 peptide therapy of NOD mouse diabetes induces a Th2 cytokine burst and downregulates autoimmunity to various beta-cell antigens. Diabetes 46: 758-764.

23. Zanin-Zhorov A, Tal G, Shivtiel S, Cohen M, Lapidot T, et al. (2005) Heat shock protein 60 activates cytokine-associated negative regulator suppressor of cytokine signaling 3 in T cells: effects on signaling, chemotaxis, and inflammation. J Immunol 175: 276-285.

24. Zanin-Zhorov A, Bruck R, Tal G, Oren S, Aeed H, et al. (2005) Heat shock protein 60 inhibits Th1-mediated hepatitis model via innate regulation of Th1/Th2 transcription factors and cytokines. J Immunol 174: 3227-3236.

25. Kol A, Lichtman AH, Finberg RW, Libby P, Kurt-Jones EA (2000) Cutting edge: heat shock protein (HSP) 60 activates the innate immune response: CD14 is an essential receptor for HSP60 activation of mononuclear cells. J Immunol 164: 13-17.

26. Flohe SB, Bruggemann J, Lendemans S, Nikulina M, Meierhoff G, et al. (2003) Human heat shock protein 60 induces maturation of dendritic cells versus a Th1-promoting phenotype. J Immunol 170: 2340-2348.

27. Ohashi K, Burkart V, Flohe S, Kolb H (2000) Cutting edge: heat shock protein 60 is a putative endogenous ligand of the toll-like receptor-4 complex. J Immunol 164: 558-561.

28. Vabulas RM, Ahmad-Nejad P, da Costa C, Miethke T, Kirschning CJ, et al. (2001) Endocytosed HSP60s use toll-like receptor 2 (TLR2) and TLR4 to activate the toll/interleukin-1 receptor signaling pathway in innate immune cells. J Biol Chem 276: 31332-31339.

29. Zanin-Zhorov A, Nussbaum G, Franitza S, Cohen IR, Lider O (2003) T cells respond to heat shock protein 60 via TLR2: activation of adhesion and inhibition of chemokine receptors. Faseb J 17: 1567-1569.

30. Sakaguchi S, Sakaguchi N, Asano M, Itoh M, Toda M (1995) Immunologic self-tolerance maintained by activated T cells expressing IL-2 receptor alpha-chains (CD25). Breakdown of a single mechanism of self-tolerance causes various autoimmune diseases. J Immunol 155: 1151-1164.

31. Dieckmann D, Plottner H, Berchtold S, Berger T, Schuler G (2001) Ex vivo isolation and characterization of CD4(+)CD25(+) T cells with regulatory properties from human blood. J Exp Med 193: 1303-1310.

32. Jonuleit H, Schmitt E, Stassen M, Tuettenberg A, Knop J, et al. (2001) Identification and functional characterization of human CD4(+)CD25(+) T cells with regulatory properties isolated from peripheral blood. J Exp Med 193: 1285-1294.

33. Sakaguchi S, Sakaguchi N, Shimizu J, Yamazaki S, Sakihama T, et al. (2001) Immunologic tolerance maintained by CD25+ CD4+ regulatory T cells: their common role in controlling autoimmunity, tumor immunity, and transplantation tolerance. Immunol Rev 182: 18-32.

34. Piccirillo CA, Shevach EM (2001) Cutting edge: control of CD8+ T cell activation by CD4+CD25+ immunoregulatory cells. J Immunol 167: 1137-1140.

35. Zanin-Zhorov A, Cahalon L, Tal G, Margalit R, Lider O, et al. (2006) Heat shock protein 60 enhances CD4+ CD25+ regulatory T cell function via innate TLR2 signaling. J Clin Invest 116: 2022-2032.
